# 

**Published:** 1861-07

**Authors:** 


### WORKS PUBLISHED DURING THE QUARTER.

### 1. *Medicine.*

ALISON.—The Physical Examination of the Chest in Pulmonary Consumption and its Intercurrent Diseases. By S. Scott Alison, M.D. Edin., F.R.C.P. Lond. With Engravings, 8vo, cloth, 12s.

ANDERSON.—Ten Lectures Introductory to the Study of Fever. By Andrew Anderson, M.D. Post 8vo, cloth, 5s.

ANDERSON.—Heads of Lectures on the Practice of Medicine, for the use of Students. By Andrew Anderson, M.D. 8vo, 2s.

BARCLAY.—Ale, Wine, Spirits, and Tobacco: a Lecture. By John Barclay, M.D., F.R.C.P. Second Edition. 8vo, sewed.

BEALE.—Lectures at the Royal College of Physicians. By Lionel S. Beale, M.D. The complete descriptive list of the specimens Illustrating the Lectures. With coloured plate, 2s.

BRINTON.—On Food and Digestion: being an Introduction to Dietetics. By W. Brinton, M.D. Post 8vo, 12s.

CHAVASSE.—Advice to a Wife on the Management of her own Health. With an Introductory Chapter especially addressed to a young Wife. By Pye Henry Chavasse, F.R.C.S. Fourth Edition, fcap. 8vo, sewed, 2s. 6d.

CHRISTISON.—On Some of the Medico-Legal Relations of the Habit of Intemperance. By Robert Christison, M.D. 8vo, sewed, 6d.

GOODFELLOW.—Lectures on Diseases of the Kidney (generally known as Bright's Disease), and Dropsy. By S. J. Goodfellow, M.D., F.R.C.P. Crown 8vo, cloth, price 7s. 6d., with Illustrations.

GULL.—An Oration delivered before the Hunterian Society, February 13th, 1861. By William W. Gull, M.D. 8vo, sewed, 1s.

HARTWIG.—A Practical Treatise on Sea-Bathing and Sea-Air. By George Hartwig, M.D. Second Edition, fcap. 8vo, sewed, 2s. 6d.

HEWITT.—On "Supporting the Perinæum;" Practical Considerations respecting the Causes and Prevention of Laceration of the Perinæum during Labour. By Graily Hewitt, M.D., M.R.C.P. 2s.

LEE.—Notes Exemplifying the State of the Medical Profession (Third Series), comprising some account of the Mismanagement of St. George's Hospital. By Edwin Lee, M.D. 8vo, sewed, 1s. 6d.

MARSH.—Instructions to Mothers and Nurses in the Lying-in Chamber. By J. C. Lory Marsh, M.D., M.R.C.P. Lond. Fcap. 8vo, sewed, 6d.

M'CORMAC.—Metanoia, a Plea for the Insane. By Henry M'Cormac, M.D. 8vo, sewed, price 1s.

M'GRIGOR.—The Autobiography and Services of Sir James M'Grigor, Bart., late Director-General of the Army Medical Department; with an Appendix of Notes and Original Correspondence. Post 8vo, cloth, 12s.

MEADOWS.—The Errors of Homœopathy. By Dr. C. J. Barr Meadows. 8vo, sewed, 1s.

MUNK.—The Roll of the Royal College of Physicians of London. Compiled from the Annals of the College, and from other authentic Sources. By William Munk, M.D., Fellow of the College, &c. &c. Volume the First, from 1518 to 1700. 8vo, 12s. cloth.

OBSTETRICAL SOCIETY.—Transactions of the Obstetrical Society of London (Second Volume) for the year 1860: with List of Officers, Fellows, &c. Plate and sixteen Woodcuts, 8vo, cloth, 15s.

PEREIRA.—Selecta e Præscriptis. By Jonathan Pereira, M.D., F.R.S. Thirteenth Edition, 24mo, cloth, 5s.

PRICE.—Scrofulous Diseases of the External Lymphatic Glands; their Nature, Variety, and Treatment: with Remarks on the Management of Scrofulous Ulcerations, Scars, and Cicatrices. By P. C. Price, F.R.C.S.E. Post 8vo, cloth, 3s. 6d.

RADCLIFFE.—On Epileptic and other Convulsive Affections of the Nervous System. Third Edition (incorporating the Gulstonian Lectures for 1860). By Charles Bland Radcliffe, M.D., F.R.C.P. Post 8vo, cloth, 7s. 6d.

RIDGE.—Ourselves, our Food, and our Physic. By B. Ridge, M.D. 12mo, cloth, 4s.

RUSSELL.—History and Heroes of the Art of Medicine. By J. R. Russell, M.D. 8vo, cloth, 14s.

RULTIMANN.—On the Therapeutic Influence of the Southern Climatic Sanatoria, particularly with reference to Chronic Tuberculosis of the Lungs. By — Rultimann, M.D. 8vo, cloth, 1s. 6d.

SIEVEKING.—On Epilepsy and Epileptiform Seizures: their Causes, Pathology, and Treatment. By Edward H. Sieveking, M.D., F.R.C.P. Second Edition, enlarged, post 8vo, cloth, 10s. 6d.

SWAYNE.—Obstetric Aphorisms, for the Use of Students commencing Midwifery Practice. By Joseph G. Swayne, M.D. Engravings on Wood. Second Edition, fcap. 8vo, cloth, 3s. 6d.

TANNER.—Manual of the Practice of Medicine. By T. E. Tanner, M.D. The Fourth Edition, enlarged and improved, 7s.

TOULMIN.—On the Importance of the Functions of the Skin in the Pathology and Treatment of Tubercular Consumption. By Abraham Toulmin, M.D., M.R.C.S.E. 8vo, sewed, 1s.

WINSLOW.—On Obscure Diseases of the Brain and Disorders of the Mind. By Forbes Winslow, M.D., D.C.L. Oxon. Second and Revised Edition, 8vo, cloth, 16s.

### 2. *Surgery.*

BROWN.—On Surgical Diseases of Women. By I. Baker Brown, F.R.C.S. Second Edition, Illustrated with Engravings, 8vo, cloth, 15s.

HEATH.—A Manual of Minor Surgery and Bandaging, for the Use of House-Surgeons, Dressers, and Junior Practitioners. By Christopher Heath, F.R.C.S. With Illustrations, fcap. 8vo, cloth, 5s.

MAUNDER.—Operative Surgery. By C. F. Maunder. With 158 Woodcuts, post 8vo, cloth, 6s.

SMITH.—Stricture of the Urethra; its Nature and Treatment in the more Severe Forms; with the Description of a New Gauge for Catheters. By Henry Smith, F.R.C.S. 8vo, sewed, price 1s.

SMITH.—Diagnostics of Aural Disease. Illustrated with a Plate and Twenty-two Wood Engravings, showing all the most important Operations performed in Aural Practice. By S. E. Smith, M.R.C.S.L. 8vo, cloth, 3s. 6d.

WATSON.—The Modern Pathology and Treatment of Venereal Diseases. By Patrick Heron Watson, M.D., F.R.C.S., Edin. 8vo, sewed, 1s.

WEBB.—The Surgeon's Ready Rules for Operations in Surgery. By Allan Webb, M.D., F.R.C.S.L. Second Edition, royal 8vo, cloth, 10s. 6d.

### 3. *Natural Science.*

BENTLEY.—A Manual of Botany. By Robert Bentley, F.L.S. Engravings on Wood, fcap. 8vo, cloth, 12s. 6d.

COBBOLD.—On the Scope, Tendency, and Educational Value of the Natural History Sciences. A Discourse delivered in the Theatre of the Royal Institution. By T. Spencer Cobbold, M.D., Lecturer at the Medical College, Middlesex Hospital. 8vo, sewed, 1s.

DALRYMPLE.—Meteorological and Medical Observations on the Climate of Egypt, with Practical Hints for Invalid Travellers. By Donald Dalrymple, M.D. Lond., F.R.C.S. Post 8vo, cloth, 4s.

PRATT.—The Genealogy of Creation, newly Translated from the Unpointed Hebrew Text of the Book of Genesis; showing the general Scientific Accuracy of the Cosmogony of Moses and the Philosophy of Creation. By Henry F. A. Pratt, M.D., M.R.C.P., Lond. 8vo, cloth, 14s.

---

### FOREIGN MEDICO-PSYCHOLOGICAL LITERATURE.

*Now ready, Second Edition, Revised, 8vo, cloth, price 16s.*

ON OBSCURE

### DISEASES OF THE BRAIN,

AND

### DISORDERS OF THE MIND.

BY FORBES WINSLOW, M.D., D.C.L. OXON.

---

CONTENTS.

CHAP. 1. Introduction.
,, 2. Morbid Phenomena of Intelligence.
,, 3. Premonitory Symptoms of Insanity.
,, 4. Confessions of Patients after recovering from Insanity; or the Condition of the Mind when in a state of Aberration.
,, 5. State of the Mind when recovering from an Attack of Insanity.
,, 6. Anomalous and Masked Affections of the Mind.
,, 7. The Stage of Consciousness.
,, 8. Stage of Exaltation.
,, 9. Stage of Mental Depression.
,, 10. Stage of Aberration.
,, 11. Impairment of Mind.
,, 12. Morbid Phenomena of Attention.
,, 13. Morbid Phenomena of Memory.
,, 14. Acute Disorders of the Memory.
CHAP. 15. Chronic (Modified) Affections of the Memory.
,, 16. Perversion and Exaltation of Memory—Memory of the Insane.
,, 17. Psychology and Pathology of Memory.
,, 18. Morbid Phenomena of Motion.
,, 19. Morbid Phenomena of Speech.
,, 20. Morbid Phenomena of Sensation.
,, 21. Morbid Phenomena of the Special Senses.
,, 22. Morbid Phenomena of Vision, Hearing, Taste, Touch, and Smell.
,, 23. Morbid Phenomena of Sleep and Dreaming.
,, 24. Morbid Phenomena of Organic and Nutritive Life.
,, 25. General Principles of Pathology, Diagnosis, Treatment, and Prophylaxis.

---

"That the Second Edition should be called for a very few months after the publication of the First is what might reasonably have been anticipated. In the present the work has been carefully revised, and many alterations and amendments have been made in it. Dr. Winslow's treatise has become the modern text-book on Insanity."—*Lancet*, 22nd June, 1861.

"Page after page is replete with curious psychological cases, which so fascinate the attention that the reader scarcely knows how to put the book down. The chapters on memory, motion, speech, and the special senses, open up phases of mental disturbance wonderful beyond even German imaginations."—*London Review*, 8th June, 1861.

---

LONDON: JOHN W. DAVIES, 54, PRINCES STREET,
LEICESTER SQUARE.

THE

### MEDICAL CRITIC

AND

### PSYCHOLOGICAL JOURNAL

EDITED

BY FORBES WINSLOW, M.D., D.C.L. OXON.

*Being a New Series of the "Psychological Journal." To be continued Quarterly, price* 3*s.* 6*d. each Number.*

---

The MEDICAL CRITIC consists of Essays, illustrative of the PRESENT and PROSPECTIVE condition of the MEDICAL PROFESSION in its MORAL, SOCIAL, POLITICAL, LITERARY, and SCIENTIFIC relations.

The Journal is enlarged to 200 pages, of which one hundred are devoted to PSYCHOLOGICAL Subjects, and the remaining portion to Articles upon general MEDICAL POLITICS, LITERATURE, and SCIENCE.

It is the object of the Editor and his Literary Staff to discuss the important Questions of the day and Books that come under review in a fair, impartial, and liberal spirit, apart from feelings of a personal, private, or party character.

---

*Contents of the First Two Numbers for* 1861:—

**No. I., for January.**

Quarterly Retrospect.
1. The Marvellous.
2. Medical Observation—Diphtheria.
3. Criminal Lunatics.
4. On the Exposition of the Principles and Details of the Syllogism.
5. Specialists and Specialities.
6. Medico-Legal Studies on the Perversion of the Moral and Affective Faculties in the precursory period of General Paralysis.
7. The Wear and Tear of Medical Life.
8. Medical Evidence—The Colney Hatch Case.
9. Maternal Love in Nature.
10. On "Non-Restraint" in the Treatment of the Insane, and the Increase of Lunatics.
11. Reason, Genius, and Madness.
12. In Memoriam: Robert Bentley Todd.
13. Medical Gossip.

Foreign Medico-Psychological Retrospect.

**No. II., for April.**

Quarterly Retrospect.
1. On the Moral Phenomena of Insanity and Eccentricity. By THOMAS MAYO.
2. The Classic Land of Suicide.
3. Professional Tricksters.
4. Quarantine and the Spread of Epidemic Diseases. By GAVIN MILROY, M.D., F.R.C.P.
5. Cottage Asylums. By W. A. F. BROWNE, Commissioner in Lunacy for Scotland.
6. Modern Surgery.
7. The Deformed and their Mental Characteristics.
8. On the Structure of the Inductive Syllogism and its Correlation to the Deductive. By R. G. LATHAM, M.D., F.R.S., &c.
9. Metanoia: a Plea for the Insane. By HENRY M'CORMAC, M.D.
10. Special Hospitals.
11. Honoraria.
12. Physiology and Legislation.
13. Miscellanea Medica.
14. The "Art of Rising" in Physic.
15. Cellular Pathology.
16. Medical Gossip.
17. Literary Gossip and Record.
18. In Memoriam: William Baly.

Foreign Medico-Psychological Literature.

---

LONDON: JOHN W. DAVIES, 54, PRINCES STREET, LEICESTER SQUARE,

*To whom Communications for the Editor and Books for Review are to be forwarded.*

